# The Book World of Medicine and Science

**Published:** 1896-05-30

**Authors:** 


### THE BOOK WORLD OF MEDICINE AND SCIENCE.

Guide to the Examination of Urine. By James Tyson, M.D., Professor of Clinical Medicine in the University of Pennsylvania. Ninth Edition. (Philadelphia: Blackiston, Son, and Co. London: Baillière, Tindall, and Cox. 1896. Price 7s. 6d.)

Dr. Tyson has in this handy little volume given a very complete and reliable account of the various processes in vogue for the examination of the urine, a much larger subject than some are accustomed to regard it. Attention is drawn to the necessity of carefully testing the urinometers used for ascertaining specific gravities, many of those sold being inaccurate. Very full directions are given of the various tests for albumin and sugar and other abnormal constituents of urine. Speaking of the quantitative estimation of albumin, the author says that "it is not unusual to overestimate the amount of albumin passed in the twenty-four hours, and thence to exaggerate the drain upon the system," and points out that supposing there be 2 per cent. of albumin, which is a very large amount, the loss will not be more than will be more than made up by half a pound of beef per week. Of course, the difficulty lies in the assimilation of this half pound of beef, but it is clear that where the digestive organs are in good order the actual loss of albumin in Bright's disease is not a very important matter. In regard to the microscopic examination, the advantages of the centrifugal machine are insisted upon. "In all instances where the sediment is scanty the urine should be centrifugated if possible." This we can fully endorse, the advantages being not merely that the examination can be completed at once and with greater efficiency, but that casts can be discovered which might become changed by decomposition had time been allowed for sedimentation. In regard to decomposition the following hint is given: "When urine is to be transported in hot weather, or kept several days for any reason, a pinch of salicylic acid added to a four-ounce vial is generally sufficient to prevent decomposition, and in no way impairs the reactions or alters the sediments." The book throughout is well printed, and the illustrations are clear and to the point.

